# Beyond Hormone Levels: Thyroid Hormone Signaling from Neurogenesis to Alzheimer’s Disease

**DOI:** 10.3390/cells15111002

**Published:** 2026-05-29

**Authors:** Cristina Del Seppia, Laura Sabatino

**Affiliations:** Institute of Clinical Physiology, National Research Council, 56124 Pisa, Italy; cristina.delseppia@cnr.it

**Keywords:** thyroid hormones, neurogenesis, neuroprotection, deiodinases, thyroid hormone receptors, thyroid hormone transporters, neurodegeneration, dementia, Alzheimer’s Disease

## Abstract

Thyroid hormones (THs) critically regulate metabolism and the central nervous system (CNS) functions, acting as key factors in neuronal differentiation, synaptogenesis and myelination. Furthermore, they play a central role in regulation of cognitive process and behavior. Therefore, compromised TH signaling can interfere with normal brain function and promote neurodegenerative progression and dementia. This review explores the role of THs from embryonic development through adulthood, with particular emphasis on their crucial role in neurogenesis. We discuss key components of TH metabolism and signaling, highlighting their neuroprotective functions in maintaining cellular homeostasis. Furthermore, we address how disruptions in TH signaling contribute to cognitive decline observed in dementia with effects that are even more pronounced in Alzheimer’s Disease (AD).

## 1. Introduction

Thyroid hormones (THs) are critical regulators of metabolic processes, growth and development across a wide variety of tissues, organs and systems, including the central nervous system (CNS) [1]. In the developing brain, THs act as temporal regulators of multiple processes, including embryonic neurogenesis, neuronal migration, differentiation, synaptogenesis and myelination, particularly in regions such as the cortex, hippocampus and cerebellum [2]. By these actions, TH play a fundamental role in shaping cognitive processes and behavior. Alterations in TH availability, both in excess and deficiency, have relevant systemic effects and developmental consequences. Hyperthyroidism, characterized by excessive TH production, leads to a hypermetabolic state characterized by high energy expenditure and heat production and is clinically associated with a variety of symptoms, including weight loss, anxiety, tremors and palpitations [3]. At the neural level, excess of TH can accelerate developmental programs, potentially leading to premature neuronal differentiation and altering timing of synaptic maturation and circuit refinement [4]. Conversely, hypothyroidism is associated with a hypometabolic state, slowed growth and development and cognitive impairment. At the developmental stage, hypothyroidism disrupts tightly regulated neurodevelopmental processes, including neuronal migration, cortical layering and myelination, ultimately resulting in structural and functional brain impairment [5]. Consistently, the clinical manifestations of hypothyroidism greatly depend on timing and severity of TH deficiency. In childhood, severe hypothyroidism can lead to cretinism, a condition classically presenting two major forms. The neurological form is characterized by irreversible CNS damage, including severe intellectual disability, deaf-mutism, spasticity and motor dysfunction, associated with early impairment of cortical and subcortical development, due to maternal and fetal hypothyroxinemia [6]. In contrast, the myxedematous form is associated with hypothyroidism beginning in late fetal or postnatal life, has a less severe degree of mental retardation than the neurological cretinism and is characterized by severe growth retardation, delayed skeletal and sexual maturation and incomplete maturation of the facial features [6]. The different phenotypes depend on the differences in the timing and duration of TH deficiency, with the early gestational deficits mainly affecting brain development, whereas later deficiency contributes predominantly to somatic growth failure. In contrast, when hypothyroidism develops in adulthood it may predispose to cognitive decline and may contribute to the worsening of neurodegenerative processes, including dementia [2].

Other relevant conditions such as congenital hypothyroidism and Allan–Herndon–Dudley syndrome further highlight the importance of TH signaling in brain development. Congenital hypothyroidism, which is prevalently caused by thyroid dysgenesis of dyshormonogenesis, represents a major cause of intellectual disability, if not promptly treated [7]. Allan–Herndon–Dudley syndrome (AHDS), a rare X-linked disorder caused by mutations in the TH transporter monocarboxylate transporters 8 (MCT8), provides an emblematic example of impaired intracellular TH signaling, despite normal or elevated levels of circulating THs, leading to severe neurodevelopmental impairment, consequent to reduced T3 availability in the brain [8,9].

Together, these conditions emphasize that both systemic and local TH availability are essential for proper brain development.

**Figure 1:**
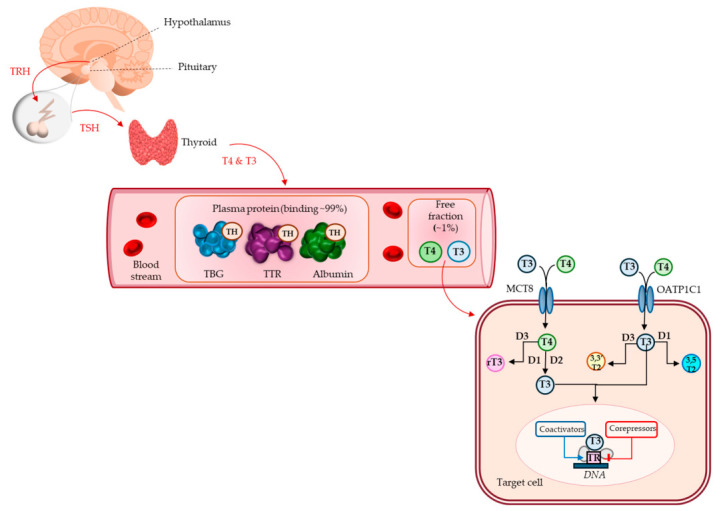
Schematic overview of TH transport, metabolism and intracellular signaling.

TH production is regulated by the hypothalamic–pituitary–thyroid axis. Thyrotropin-releasing hormone, synthesized by neurons in the hypothalamic paraventricular nucleus, stimulates the thyrotrope cells in the anterior pituitary gland to secrete thyroid-stimulating hormone which, in turn, regulates thyroid growth and thyroid hormone synthesis. Thyroxine (T4) is the primary product of the thyroid gland, accounting for approximately 80% of total TH production, whereas triiodothyronine (T3) represents the remaining 20% [10,11]. In circulation, most THs are bound to plasma proteins, including thyroxine-binding globulin, transthyretin, and albumin, with only a small fraction existing in a free form [1]. Upon entering target cells through specific membrane transporters, T4 can be converted to the more active T3 by type 1 or type 2 deiodinases (D1 and D2) or inactivated to reverse-T3 (rT3) by type 3 deiodinase (D3) [12,13]. T3, although secreted in smaller amounts by the thyroid, is largely generated in peripheral tissues via T4 deiodination and represents the biologically active form of THs, being approximately three times more potent than T4 [14]. Like T4, T3 circulates predominantly bound to plasma proteins, with only the free fraction able to enter cells through specific transporters. Once inside the cell, T3 binds TH receptors (TRs) in the nucleus with higher affinity than T4, thereby regulating gene transcription and mediating most of the physiological effects of TH. Intracellular T3 availability is controlled by deiodinases, with D2 promoting local T3 production and D3 contributing to its inactivation to 3,3′-diiodothyronine (3,3′-T2). Both TR isoforms TRα and TRβ are differently expressed across distinct regions of the CNS, contributing to neuronal development and function. TRα1 is the predominant subtype in the brain and mediates most of the maturation and cognitive processes [15,16,17]. In Figure 1, a schematic overview of TH transport, metabolism and intracellular signaling is shown.

The HPT axis regulates systemic TH production. Hypothalamic TRH stimulates pituitary secretion of TSH, which in turn promotes thyroidal release of T4 and T3. In the bloodstream, nearly 99% of circulating thyroid hormones are bound to plasma transport proteins, mainly TBG, TTR and albumin, whereas only a small free fraction is biologically available. Free T4 and T3 enter target cells through specific membrane transporters, such as TH transporter MCT8 and organic anion-transporting polypeptide 1c1 (OATP1C1). Within cells, local TH availability is tightly regulated by deiodinases: D2 converts T4 into the active hormone T3, whereas D3 catalyzes hormone inactivation by converting T4 into rT3 and T3 into T2. D1 also contributes to peripheral T3 production and T3 metabolism. Intracellular T3 binds TRs in the nucleus, modulating gene transcription through the recruitment of coactivators or corepressors and thereby regulating thyroid hormone-responsive genes.

HPT: hypothalamic–pituitary–thyroid axis; TRH: thyrotropin-releasing hormone; TSH: thyroid-stimulating hormone; TBG: thyroxine-binding globulin; TTR: transthyretin.

The accumulated evidence suggests that altered thyroid status may contribute to cognitive decline and dementia. Disruption of TH signaling, even in the presence of normal circulating hormone levels, can impair brain plasticity and exacerbate neurodegenerative processes, such as amyloid-β (Aβ) accumulation, which disrupts synaptic function, impairs neuronal communication, and triggers inflammatory responses [18]. The accumulation of Aβ is often accompanied by tau protein hyperphosphorylation, neurofibrillary tangle formation and neuronal loss, all considered pathological hallmarks of dementia in general and, more specifically, of Alzheimer’s Disease (AD) [2]. Collectively, these findings support the hypothesis that TH dysregulation may represent both a potential biomarker and a potentially treatable risk factor for dementia. Moreover, data on expression and activity of TH transporters and deiodinases in many areas of the brain suggest that there is a tissue-specific regulation of TH levels and that serum TH concentrations may not be unequivocally representative of hormonal status in the tissues [19,20]. These considerations automatically bring into question the effectiveness of TH substitutive therapy in hypothyroid patients to restore T3 levels in the brain. The cellular mechanisms linking hypothyroidism to neurodegeneration remain to be fully understood, as does the cell signaling underlying an effective compensation of T3 deficiency. In the present review, we aim to provide an integrated overview of TH signaling across the lifespan, from neurodevelopment to adult neurogenesis, and to discuss how its dysregulation contributes to cognitive decline and neurodegenerative processes, with particular emphasis on AD.

## 2. Multilevel Regulation of TH Signaling in the CNS

Traditionally, TH actions were thought to be regulated primarily through the modulation of circulating free hormone levels, which were intracellularly activated and exerted their functions in the nucleus after binding TRs. More recently, this classical view has been revised, as evidence has emerged that regulatory mechanisms acting at multiple levels concurrently influence TH availability and activity. At the cellular level, THs exert both genomic and nongenomic effects [21,22,23]. Genomic actions occur when the biologically active T3 binds nuclear TRs. In contrast, nongenomic actions are mediated independently of nuclear TRs and typically require the interaction of T4 with the plasma membrane integrin αvβ3 receptor, leading to the activation of distinct intracellular signaling pathways [24,25]. Importantly, the genomic and nongenomic mechanisms of TH action do not always represent mutually exclusive processes but can arise sequentially or simultaneously [26].

TH regulation takes place at both central (within the CNS) and peripheral levels. Moreover, multiple mechanisms in cells and tissues are highly dynamic, involving several signaling pathways that can act independently of variations in TH circulating levels [27]. Local TH bioavailability is regulated by the activity of specific TH transporters, deiodinase enzymes and TRs. These components are considered key factors of the mechanisms used by the cells to uncouple intracellular TH concentrations from circulating levels, thereby modulating the local response. TH transporters mediate the TH uptake across the cytosolic membrane [28]. Many TH transporters have been identified at the molecular level, but only a few are classified as specific TH transporters, including MCT8 and MCT10, L-type amino acid transporter 1 and 2, and OATP1C1 [29,30]. In particular, MCT8 mediates the transport of T4, T3 and rT3, whereas MCT10 transports T4 and T3. Notably, these transporters are bidirectional, mediating both influx and efflux of TH, thus enabling the rapid establishment of hormonal equilibrium [29].

As previously mentioned, the relevance of TH transport is tightly associated with the discovery that mutation of MCT8 causes Allan–Herndon–Dudley syndrome (AHDS) [8]. Moreover, the expression of MCT8 at the blood–brain barrier (BBB) level also suggests its involvement in the T3 uptake at this site [31]. In MCT8-deficient mice, high serum levels of T3 and normal/low serum levels of T4 were observed, whereas brain uptake of both hormones was diminished, suggesting that MCT8 is responsible for TH transport across BBB in mice [32].

Furthermore, the observation that brain development and function are normal in D2-deficient mice suggests that circulating T3 levels can compensate for the lack of T3 generation from intracellular deiodination of T4 and, more importantly, that T3 can be directly taken up from the circulation [33]. Upon entering target cells, T4 can be converted to T3 by D1 or D2 or inactivated to rT3 by D3, whereas T3 can be further metabolized to inactive metabolites such as T2 [12,13]. The three enzymes are synthesized by three different genes and their active sites contain a selenocysteine amino acid, crucial for enzymatic activity [34]. D1 is the only enzyme capable of catalyzing both outer- and inner-ring deiodination of TH, whereas D2 catalyzes exclusively outer-ring deiodination and D3 is restricted to inner-ring deiodination [35]. The deiodinases are membrane-anchored homodimers and have different location in the cells, being D1 and D3 at cell membrane, whereas D2 is sublocalized in the endoplasmic reticulum membrane. The three enzymes are expressed ubiquitously throughout development and in a wide range of tissues [36]. D2 is mainly responsible for local production of T3 in the tissues and its activity increases in hypothyroid conditions, whereas in hyperthyroidism it is inactivated by selective ubiquination [37]. In the brain, D2 plays a central role in T3 production and the principal sites for D2 activity are the glial cells: the tanycytes, lining the third ventricle surface, and the astrocytes, distributed throughout the brain. *D2* mRNA has been detected both in the glial cell body and in their cellular processes, supporting the existence of a close astrocyte–neuron interaction in the maintenance of cerebral TH homeostasis. After crossing the BBB, circulating T4 is taken up by astrocytic end-feet in contact with capillaries, converted into T3 by D2 and subsequently delivered to neighboring neurons which, therefore, rely largely on glial-derived T3 for proper TH signaling [38,39]. The third deiodinase, D3, is the enzyme responsible for the physiological inactivation of TH. It is preferentially expressed in embryonic tissues, brain and placenta but in some pathological conditions it can also be activated in other tissues [40].

TRs mediate genomic actions of THs and are ligand-activated factors which directly interact with specific sequences in the promoters of target genes, called TH response elements (TREs). By modulating the transcription of target genes in response to ligand binding, TRs play a crucial regulatory role in TH signaling, thereby maintaining proper cellular functions and activities [41]. Notably, TRs can also act independently of ligand binding. When unliganded, they are still able to bind TREs but preferentially recruit corepressors, mediating transcriptional repression [23]. This feature of TRs has important functional implications, since it allows different tissues to respond differently, according to local TH levels [42]. Furthermore, TR functions are not confined to the nucleus; in fact, they are dynamic proteins that can rapidly shuttle from their main nuclear localization to the cytoplasm where they may exert important cytoplasmic effects [43]. The finely balanced shuttling of TRs between the nucleus and the cytosol is considered an important regulatory checkpoint for the expression of TH-responsive genes. There are two TR genes, *THRA* and *THRB*, and each one encodes distinct variants. TRα1 is the predominant subtype in the brain and mediates most of the maturation and cognitive processes [17]. TRβ variants are expressed later during development and in a more restricted set of tissues; notably, they are present in sensory neurons [44,45].

## 3. TH and the CNS: From Embryonic Development to Adulthood

The thyroid gland is among the earliest endocrine organs to emerge during embryonic development, and its tightly regulated ontogeny is fundamental for the establishment of TH signaling in the developing brain. In humans, thyroid morphogenesis is initiated around gestational days 20–24, when an endodermal thickening appears at the base of the tongue. This structure subsequently forms the thyroid primordium, which descends along the midline of the neck via the thyroglossal duct and reaches its definitive pretracheal position, assuming its bilobed shape by approximately weeks 7–10 of gestation. During this migratory and organizational phase, a coordinated transcriptional program, principally involving *NKX2-1*, *PAX8*, and *FOXE1* genes, ensures proper specification and differentiation of thyroid follicular cells [46].

Functional differentiation of the thyroid gland follows structural development, with the onset of TH synthesis occurring around the 10th–12th week of gestation. This period represents a key transition in the developmental process as the fetal thyroid gland begins to concentrate iodine and refines the biochemical machinery required for TH synthesis. However, since the fetal thyroid becomes functionally autonomous and capable of TH synthesis under fetal TSH only toward the middle of pregnancy (18–20 weeks), maternal THs remain an important source during early and mid-gestation. The gradual shift to fetal thyroid autonomy is particularly relevant for brain development, as it coincides with crucial neurodevelopmental events including neuronal proliferation, migration, and early differentiation [47,48,49].

In parallel with these processes, TH signaling in the developing CNS becomes increasingly prominent. The effectiveness of maternal TH action in the fetus is ensured by the early expression of TRs, deiodinases, and TH transporters, which together allow spatial and temporal regulation of TH availability during neurodevelopment [44,50]. TRα1 is the predominant form also in the developing brain, suggesting an early ability of neural tissue to respond to THs. In parallel, deiodinases are regulated in a time- and region-specific manner: D3 is highly expressed in early gestation to limit the intracellular levels of T3 and protect the embryo from excessive hormonal exposure, whereas D2 expression increases later in gestation to promote local T3 increase, thus favoring TH signaling during active neuronal differentiation and circuits formation.

The relevance of this tightly regulated timing is underlined by clinical and experimental evidence associating early TH deficiency with severe neurodevelopmental impairments [51,52,53]. For example, congenital hypothyroidism and endemic cretinism are conditions that arise from disruptions of thyroid–brain axis maturation, particularly when hormone deficiency occurs during early gestation, when maternal supply is still critical [54,55]. Together, these observations highlight the essential role of precise temporal coordination between thyroid gland development and the onset of TH signaling in the brain, a process that is fundamental for normal human neurodevelopment.

In the adult brain, THs contribute to the regulation of multiple key processes, including synaptic plasticity, dendritic spine remodeling and myelin integrity, all of which are essential to maintain efficient neuronal network functions. Furthermore, THs regulate glial cell activity, glucose utilization in the brain and the balance of principal neurotransmitter systems, including serotoninergic, noradrenergic and GABAergic pathways, thus playing an important role in modulation of cognitive and behavioral functions [56]. Consistent with these roles, alterations in TH signaling in adulthood are associated with cognitive dysfunction, memory impairment and psychomotor slowing. However, these cognitive and behavioral deficits observed in TH alterations are often associated with neuropsychiatric manifestations, including depression symptoms, making it difficult to attribute these deficits solely to TH deficiency. For instance, patients with Resistance to Thyroid Hormone α (RTHα), a rare genetic disorder caused by mutations in the *THRA* gene encoding TRα, despite near-normal circulating TH levels, exhibit tissue-specific hypothyroid features. In RTHα neurocognitive impairment, delayed psychomotor development and behavioral abnormalities are not directly associates with TH deficiency but with compromised TRα-mediated signaling [57].

## 4. THs and Adult Neurogenesis

In the last decade, the role of THs as key regulatory factors of neurogenic processes in adults has emerged. The hippocampus shows substantial morphological plasticity throughout life and is one of the few regions in the brain with the ability to generate new neurons throughout adult life in several mammalian species including humans. These newborn neurons play a central role in the hippocampal function [58].

Two main neurogenic niches containing a progenitor population have been categorized in mammalian brains: the subventricular zone (SVZ), lining the lateral ventricles [59], and in the subgranular zone (SGZ) of the dental gyrus subfield of the hippocampus [60]. Other less characterized niches have also been described in the hypothalamus, in the striatum, in the cerebral cortex and the amygdala [60,61,62,63]. Although adult neurogenesis in the SGZ and SVZ shares many common features, the lineage potential and ultimate outcome of the neurogenic process in these two regions are distinct.

Most research exploring mechanisms underlying TH regulatory activity on neurogenesis has been conducted in rodents. Progenitor cells retain the capacity to proliferate, undergo maturation and integrate in neuronal circuits. All of these stages are regulated by niche-mediated factors, including hormones, neurotransmitters and growth factors [64,65,66]. Many studies evidenced important effects of THs on progenitor development in these neurogenic niches. Experimental approaches were mainly based on modulation of TH status [67], evaluation of TR isoform mutation [68] and direct in vitro stimulation by THs [69,70]. In the adult neurogenic niche, the major players of TH signaling (TH transporters, TH deiodinases and TRs) are all expressed and impact the way THs can influence distinct stages of adult neurogenesis [71]. Local TH availability within the niches is tightly controlled by TH transporters and deiodinases, which finely regulate intracellular T3 signaling in a stage-specific manner. In particular, D2-mediated T4 to T3 conversion promotes intracellular T3 availability, whereas D3 limits T3 action through hormone inactivation, thus contributing to the fine regulation of progenitor proliferation and differentiation.

Studies in the adult mouse SVZ showed that only the TRα1 isoform is present; however, it is not detectable at the neural stem cell (NSC) level; it starts to be detected in early progenitors and is maintained in mature neuroblasts. T3/TRα1 signaling plays an important role in adult neurogenesis, acting as a direct transcriptional repressor that permits the switch that activates NSCs commitment towards migrating neuroblast phenotype. Among these genes, *SOX2* is particularly relevant since it is involved in the control of neural progenitor maintenance and its T3/TRα1-mediated repression permits the transcription of proneural factors. Inversely, TRα1 loss of function increases the numbers of the NSC population and decreasing neuroblast numbers [72]. Furthermore, in the presence of T3, TRα1 is thought to repress gene transcription of cell cycle genes *Cyclin D1* and *c-myc*, thereby accelerating cell cycle exit and the progression to neuroblast maturation [73]. Several T3-responsive genes and molecular pathways have been implicated in key steps of adult neurogenesis. For instance, genes involved in neuronal differentiation, maturation and synaptic plasticity, such as *NeuroD1*, doublecortin (*DCX*), and *Reelin*, are positively regulated by T3 signaling, promoting neuronal lineage commitment and migration [69,74]. Additionally, T3 regulates the expression of survival-associated factors, including brain-derived neurotrophic factor (*BDNF*) and nerve growth factor (*NGF*), thereby influencing progenitor proliferation and neuronal survival [74]. However, the molecular mechanisms that underlie these neurogenic events and the specific target genes involved, as well as their possible link to the regulation of learning, memory and mood processes in the adult, remain to be fully elucidated.

Although several studies showed that altered T3 levels influence proliferation, survival and differentiation in the cells of neurogenic niches, limited data are available on the effects of T3 on the precise stages of adult progenitor development. In vivo studies indicated a T3-induced proliferative activity on SVZ progenitors, whereas in the SGZ, T3 is believed to predominantly act on post-mitotic progenitors, determining the cell cycle exit and differentiation [73,75]. Adult-onset hypothyroidism leads to reduced survival and differentiation of hippocampal progenitor cells and this effect can be fully reversed by restoring euthyroid status, indicating that it is induced by decreased TH levels [70]. In contrast, no alteration in hippocampal progenitor proliferation or differentiation was observed in adult hyperthyroid rats, suggesting that the effects of THs on adult progenitor cells may already be fully supported when hormone levels are normal or higher [67,70].

**Figure 2:**
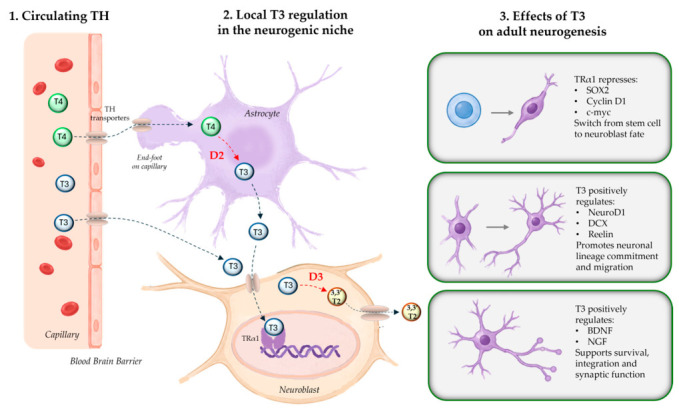
TH regulation in adult neurogenesis. (1) Circulating T4 and T3 cross the blood–brain barrier through specific TH transporters and reach the neurogenic niche. (2) Local TH availability is tightly regulated by astrocytes and neural precursor cells through the activity of deiodinases. D2, mainly expressed in astrocytes, converts T4 into biologically active T3, increasing local hormone availability, whereas D3 inactivates T3 and limits TH signaling in neuroblasts. Intracellular T3 binds TRα1 to regulate transcriptional programs involved in neuronal differentiation. (3) T3 promotes adult neurogenesis by modulating sequential developmental stages. TRα1 represses *SOX2*, *CyclinD1* and c-myc, favoring the transition from stem cell maintenance to neuroblast commitment. T3 further enhances the expression of genes involved in neuronal lineage progression and migration, including *NeuroD*, *DCX* and *Reelin*, and supports neuronal survival, integration and synaptic function through the regulation of neurotrophic factors such as BDNF and NGF.

Overall, these findings support the hypothesis that T3 acts as a fine regulator of adult neurogenesis by integrating systemic hormonal signals with local specific regulation. Future studies aim to better define T3-dependent transcriptional networks and their temporal dynamics across different progenitor stages. In Figure 2, TH regulation in adult neurogenesis is shown.

## 5. THs and Dementia: Epidemiological Evidence and Mechanistic Insights

The relationship between thyroid status and cognitive health is characterized by considerable complexity. Numerous studies suggest a robust link between thyroid abnormalities and cognitive decline [76]. Specifically, an increased risk of cognitive impairment has been identified across the full spectrum of thyroid dysfunction: clinical and subclinical hyperthyroidism, as well as clinical and subclinical hypothyroidism [77]. However, clinical evidence remains heterogeneous and sometimes contradictory, complicating the distinction between simple association and direct causation. 

Recent systematic reviews and meta-analyses point to thyroid dysfunction as a risk factor for neurodegeneration. A meta-analysis of 15 studies indicated that, in longitudinal settings, both overt and subclinical hyperthyroidism significantly increase the risk of dementia [78]. This is supported by Ye et al. [79], whose review of 12 studies focusing on various dementia types concluded that hyperthyroid states, regardless of clinical severity, are associated with elevated risk.

Regarding the evidence linking hypothyroidism and dementia, we report two large-scale investigations are particularly noteworthy: a Danish national database study and a cohort study involving 15,686 patients. Both identified an increased risk of dementia among hypothyroid subjects. Specifically, Thvilum et al. (2021) reported a 22% increased risk [80], while Wieland et al. (2022) found that this risk increased to 81% in untreated patients over 64 years of age [81].

These findings are supported by a recent systematic review of nine studies, six of which demonstrated a robust and significant association between hypothyroidism and dementia, although the authors conclude that the relationship between thyroid dysfunction and cognitive impairment remains complex and ambiguous [82]. Zhu and co-authors [83] suggest that the association between hypothyroidism and cognitive impairment may be more pronounced in patients with mild cognitive impairment (MCI) than in those with advanced AD or established dementia. In addition, some evidence suggests that subclinical hypothyroidism, characterized by mild elevations of thyroid-stimulating hormone (TSH), may be associated with increased longevity and higher cognitive scores in the very elderly [84]. Furthermore, the clinical utility of hormone replacement therapy remains a matter of debate. A landmark randomized trial concluded that levothyroxine treatment provided no significant clinical benefit regarding cognitive function or hypothyroid symptoms in older adults with subclinical hypothyroidism [85].

The heterogeneity emerging from recent literature necessitates caution. Several critical factors may confuse the true relationship between the thyroid and the brain. A primary concern is that the physiological stress inherent in early neurodegeneration may alter TSH and free thyroxine (FT4) levels, rather than thyroid dysfunction being the primary driver of the brain pathology [81]. Furthermore, circulating hormone levels often do not reflect the internal microenvironment of the CNS. As discussed in Section 2, the brain regulates local hormone availability through specific transporters and local deiodinase activity. These local mechanisms can mask profound tissue deficiencies even in patients who appear biochemically “euthyroid” according to peripheral blood parameters [38].

The mechanistic pathways linking THs to dementia are multifactorial. THs are critical regulators of cellular metabolism and energy production. In particular, T3 plays a key role in modulating intracellular lipid accumulation [86]; consequently, reduced hormone levels may impair lipid availability at the cellular level [87]. Recent positron emission tomography (PET) imaging has revealed reduced glucose metabolism in hypothyroid subjects across several brain regions, including the bilateral amygdala, hippocampus, and the perigenual and posterior anterior cingulate cortex. Such metabolic deficits are likely to disrupt neural circuits, impair synaptic plasticity and promote neuroinflammation, thereby compromising memory, learning, motor coordination and emotional regulation [88].

Furthermore, THs appear to exert a protective effect on the BBB. Hormone deficiency may compromise BBB integrity, facilitating the influx of toxins and pro-inflammatory cytokines into the brain, which triggers the neuroinflammatory cascades underlying neurodegenerative disease [89].

THs are also essential for maintaining brain homeostasis as their deficiency leads to elevated oxidative stress [90]. In addition, THs govern neuronal growth, myelination, and neurotransmitter synthesis. Their deficiency can result in the aberrant development of axons and myelin sheaths, further predisposing individuals to cognitive impairment and progression to dementia [76].

## 6. THs in AD: Molecular Determinants of Neurodegeneration

AD is a complex multifactorial disorder characterized by a network of implicated proteins [91,92]. The most extensively studied mechanisms involve the extracellular accumulation of Aβ plaques and the formation of intracellular neurofibrillary tangles (NFTs) composed of hyperphosphorylated tau protein [93].

Recent evidence suggests that TH deficiency may exacerbate these pathologies by upregulating the expression of the Amyloid Precursor Protein (APP) and facilitating the aggregation of both Aβ plaques and tau aggregations [94].

Peptidyl-prolyl cis/trans isomerase 1 (PIN1) is a key enzyme that regulates the conformation of the amyloid precursor protein (APP). By binding to phosphorylated Ser/Thr-Pro residues, PIN1 catalyzes the transition of APP from the cis to the trans form, the latter preferred by secretases for the non-amyloidogenic pathway [95,96].

In AD patients, PIN1 activity is significantly reduced, favoring the accumulation of the pathogenic cis form [97]. THs, particularly T3, play a crucial neuroprotective effect by regulating mitochondrial biogenesis and the efficiency of the electron transport chain, mitigating oxidative stress [98,99]. In the absence of T3, the increase in reactive oxygen species (ROS) and the concomitant reduction in ATP render neurons vulnerable to apoptosis. Furthermore, the oxidative environment induces the post-translational deactivation of PIN1, blocking the conversion of APP into trans isoform and thus accelerating the process of amyloidogenesis [90].

Adequate TH levels are essential for metabolic homeostasis, neuroprotection, and synaptic plasticity. Reduced TH signaling leads to the downregulation of brain-derived neurotrophic factor (BDNF) and Reelin, both of which are essential for neuronal plasticity [74]. Deficiencies of these TH-dependent factors have been associated with AD [100,101].

Additionally, THs regulate genes involved in the microglial response. In particular, it has been observed that TH deficiency can lead to the overexpression of proinflammatory cytokines such as *TNF-α* and *IL-1β* [102]. Recent work has shown that hypothyroidism promotes polarization toward proinflammatory microglia (M1) by inhibiting the expression of *BDNF*, *IL-10*, and *Arg1* in the brain [103].

The direct regulation of genes involved in Aβ production and clearance by THs is remarkable. In the (5xFAD) experimental model, T3 was shown to directly influence *APP* gene expression and subsequent secretase-mediated cleavage. In adult mice, a three-week hypothyroid state resulted in elevated *APP* mRNA levels, whereas hyperthyroidism led to a reduction [104]. These findings are supported by studies in murine neuroblastoma cells, where T3 negatively regulates *APP* transcription by promoting the binding of the TRs to DNA sequences within the first exon of the gene. In hypothyroidism, this repression is lost, leading to *APP* overexpression [105]. An increase in APP peptides may derive from an alteration in protein production or in the enzymes responsible for APP cleavage.

Indeed, it has been observed that THs influence the activity of secretases, particularly BACE1. In the presence of normal T3 levels, the α-secretase isoform is activated and functions correctly, preventing the formation of the amyloidogenic peptide. However, in hypothyroidism, the balance shifts toward the β-secretase isoform, leading to an increase in APP peptides [106].

Crucially, THs are involved in the degradation of amyloidogenic peptides. THs increase the expression of LRP1, the primary endothelial receptor responsible for the abluminal-to-luminal transcytosis of Aβ across the blood–brain barrier. Conversely, TH deficiency reduces LRP1 levels, impairing Aβ transport from the brain to the periphery [107,108]. Furthermore, T3 stimulates the production of amyloid-degrading enzymes, such as the neutral endopeptidase neprilysin (NEP) [109]. It has been observed that in hypothyroid states, reduced *NEP* expression is correlated with a higher density of Aβ plaques in the cerebral cortex [110]. Other genes involved in Aβ clearance modulated by TH include *APOE*, *LRP1*, *TREM2*, *AQP4*, and *ABCB1* [111].

Simultaneously, TH deficiency increases the activity of kinases such as glycogen synthase kinase 3 (GSK3), which hyperphosphorylate tau [90]. Hyperphosphorylated tau loses its affinity for microtubules, leading to their disassembly and the formation of toxic intracellular tangles [112]. The resulting loss of microtubule stability disrupts axonal transport of essential nutrients and signaling molecules, ultimately causing dysfunction in cellular homeostasis [113]. Finally, TH-modulated secretase activity may contribute to the cognitive deficits observed in AD through the N-cadherin pathway. The secretase complex cleaves N-cadherin to produce a cytosolic fragment, N-Cad/CTF2 [114,115]. This fragment normally promotes the proteolysis of the CREB-binding protein (CBP) [116]. CBP is a nuclear co-activator for the transcription factor cAMP Response Element-Binding protein (CREB), and their interaction is essential for memory-related gene expression. In AD, altered secretase activity leads to reduced CTF2, resulting in abnormal accumulation of CBP. This excess CBP interacts with CREB in a dysregulated manner, causing aberrant gene activation. These mechanisms are relevant, as they may contribute to exacerbate cognitive decline in AD [117]. Table 1 summarizes the principal molecular targets and biological effects associated with TH deficiency in neurodegeneration, as described in the text.

## 7. TH and Therapeutical Hypothesis in AD

The identification of effective pharmacological strategies for AD remains one of the major challenges in pharmacotherapy research. The complexity of AD, resulting from the interplay of multiple pathological processes, makes it particularly difficult to identify compounds able to reliably restore cellular homeostasis. AD is a complex multifactorial disorder at all stages of its progression. Therefore, therapeutic approaches that target a single mechanism are likely to be ineffective or only partially effective.

Numerous studies have identified key pathological factors characterizing the disease, such as Aβ deposition as plaques in the neocortex and intracellular aggregates of hyperphosphorylated tau protein in limbic and cortical association areas, which often occur long before the onset of cognitive impairment. However, considerable uncertainty remains about the events that predispose individuals to rapid cognitive decline, which, in turn, complicate the individuation of adequate prevention and treatment strategies.

Advanced age, genetic background, inflammation, metabolic and oxidative stress, all contribute to impaired cellular signaling. Altogether, these aspects suggest that effective therapeutic strategies should target multiple pathways involved in disease progression [118]. Therefore, the predominant focus on monotherapy trials—such as cholinesterase inhibitors or memantine—may, at least in part, explain the current lack of effective therapeutic options [119].

In this context, THs have emerged as a compelling area of study. THs are fundamental for the proper formation and development of CNS, from embryonic development throughout the entire lifespan. Population-based studies suggested the existence of a close relationship between TH level alterations and the risk of AD. Hypothyroidism becomes more prevalent with age and, if untreated, is frequently associated with learning and memory impairments [120]. However, the efficacy of TH replacement in hypothyroid patients remains controversial. In some cases, in fact, individuals continue to show symptoms of cognitive dysfunction, despite normal levels of circulating THs. This may suggest that serum TH concentrations do not necessarily mirror hormone availability in the target tissues, where local TH content and activity is strictly regulated by hormone transporters and deiodinases [38].

Experimental studies in AD animal models support the hypothesis that TH signaling may exert neuroprotective effects. L-T4 administration in Aβ-induced AD mouse models ameliorate cognitive impairment, reduce oxidative stress and protect hippocampal neurons from apoptosis, suggesting that THs may counteract several pathological mechanisms associated with neurodegeneration [121]. Furthermore, the administration of Aβ impairs the neuronal electrophysiological spontaneous activity in the hippocampus, and L-T4 administration restores spontaneous hippocampal neuronal activity, thus protecting neuronal circuits from Aβ-induced toxicity [122].

Nevertheless, the currently available evidence is still largely limited to preclinical models and the translation of TH-based therapies to humans remains extremely complex. So far, no relevant clinical trials have demonstrated clear cognitive benefits of TH supplementation or thyromimetic treatment in patients with AD. Moreover, systemic TH administration may induce important peripheral adverse effects, particularly at cardiovascular and skeletal levels, thus discouraging its therapeutic applicability. Another major limitation concerns the difficulty of selectively enhancing TH signaling in the brain without altering peripheral hormone homeostasis. Therefore, although TH-based approaches represent an interesting therapeutic hypothesis, additional studies are needed to better define effective targets, treatment windows and CNS-selective compounds before clinical translation can be realistically considered.

## 8. Conclusions

The growing understanding of TH signaling highlights a complex regulatory network operating at multiple levels. It is now clear that TH availability in the brain is not simply a reflection of circulating blood levels but is tightly regulated by local mechanisms, including specific transporters, deiodinase enzymes and dynamic modulation of TRs.

The relationship between THs and the CNS is multifaceted, including critical roles during both development and adulthood. In development, THs are essential for structural assembly, neuronal migration and synaptogenesis. In adulthood, they play important roles in maintaining metabolic homeostasis, neuroprotection and regulating adult neurogenesis within specific niches.

The link between hypothyroidism and dementia is well known, and clinical evidence strongly suggests that untreated hypothyroidism is a significant risk factor for cognitive decline. In adults, TH deficiency contributes to neurodegeneration by reducing glucose metabolism, increasing oxidative stress, and compromising the integrity of the BBB. Furthermore, TH deficiency may contribute to the development of AD through two main mechanisms. First, the loss of TH-mediated repression promotes amyloidogenesis, by increasing APP production and enhancing β-secretase activity toward the pro-amyloidogenic pathway. Second, the deficiency of TH-activated kinases such as GSK3 promotes tau protein hyperphosphorylation and microtubule destabilization. Despite extensive research into potential therapeutical strategies, an effective cure for AD remains elusive and many promising new drugs have failed to achieve significant endpoints over the last decades.

Given the multiple pathological mechanisms underlying the onset and progression of AD, a combined, multitargeted therapeutic strategy might represent the most promising approach in the future. In this context, recognizing THs as a central regulator of cellular homeostasis offers new perspectives for intervention, suggesting that maintaining local hormonal balance may be critical in preventing or slowing neurodegenerative decline.

Future research will be essential to deepen our understanding of processes involving THs in AD and to translate this knowledge into effective therapeutic strategies.

## Figures and Tables

**Table 1:** Impact of TH deficiency on neurodegenerative and AD-related processes (upregulation: upward arrow; downregulation: downward arrow).

Process	Specific Target	TH Deficiency		References
Aβ Production	*APP*	expression	**↓**	[94]
Aβ Clearance	*LRP1*, *ABCB1*, *PIN1*, *BACE1*	expression/protein activity	**↓**	[97,106,108,111]
Aβ Degradation	NEP	protein level	**↓**	[109]
Neuroprotection	*BDNF/Reelin*	expression/protein activity	**↓**	[100,101]
Inflammation	*TNF-α*, *IL-1β*	expression/protein activity	**↑**	[102]
Inflammation	*BDNF*, *IL-10*, *Arg1*	expression	**↓**	[103]
Homeostasis	*TREM2*, *APOE*, *AQP4*	expression	**↑**	[111]
Tau Phosphorylation	*GSK3*	expression	**↑**	[90]
Genes activation	N-Cad secretase	protein level	**↓**	[117]

## Data Availability

No new data were created or analyzed in this study.

## Abbreviations

The following abbreviations are used in this manuscript:

AHDS	Allan–Herndon–Dudley syndrome
AD	Alzheimer’s Disease
APP	Amyloid Precursor Protein
Aβ	Amyloid-β
BBB	Blood Brain Barrier
CREB	cAMP Response Element-Binding protein
CNS	Central Nervous System
CBP	CREB-Binding Protein
D1	Deiodinase 1
D2	Deiodinase 2
D2	Deiodinase 3
GSK3	Glycogen Synthase Kinase 3
MCT8	Monocarboxylate Transporters 8
MCT10	Monocarboxylate Transporters 10
NEP	Neutral Endopeptidase Neprilysin
NFTs	Neurofibrillary Tangles
NSCs	Neural Stem Cells
OATP1C1	Organic Anion-Transporting Polypeptide 1c1
PIN1	Peptidyl-prolyl cis/trans Isomerase 1
ROS	Reactive Oxygen Species
rT3	Reverse T3
SGZ	Subgranular Zone
SVZ	Subventricular Zone
TRs	TH Receptors
TH	Thyroid Hormones
TREs	Thyroid Response Elements
T4	Thyroxine
T3	Triiodothyronine
